# Disrespect and abuse of women during the process of childbirth in the 2015 Pelotas birth cohort

**DOI:** 10.1186/s12978-018-0495-6

**Published:** 2018-03-27

**Authors:** Marilia Arndt Mesenburg, Cesar Gomes Victora, Suzzane Jacob Serruya, Rodolfo Ponce de León, Andrea Homsi Damaso, Marlos Rodrigues Domingues, Mariangela Freitas da Silveira

**Affiliations:** 1 Post-Graduate Program in Epidemiology, Federal University of Pelotas, Pelotas, Brazil; 2 Latin American Center of Perinatology, Women and Reproductive Health, Montevideo, Uruguay; 3 Post-Graduate Program in Physical Education, Federal University of Pelotas, Pelotas, Brazil

## Abstract

**Background:**

The disrespect and abuse of women during the process of childbirth is an emergent and global problem and only few studies have investigated this worrying issue. The objective of the present study was to describe the prevalence of disrespect and abuse of women during childbirth in Pelotas City, Brazil, and to investigate the factors involved.

**Methods:**

This was a cross-sectional population-based study of women delivering members of the 2015 Pelotas birth cohort. Information relating to disrespect and abuse during childbirth was obtained by household interview 3 months after delivery. The information related to verbal and physical abuse, denial of care and invasive and/or inappropriate procedures. Poisson regression was used to evaluate the factors associated with one or more, and two or more, types of disrespectful treatment or abuse.

**Results:**

A total of 4275 women took part in a perinatal study. During the three-month follow-up, we interviewed 4087 biological mothers with regards to disrespect and abuse. Approximately 10% of women reported having experienced verbal abuse, 6% denial of care, 6% undesirable or inappropriate procedures and 5% physical abuse. At least one type of disrespect or abuse was reported by 18.3% of mothers (95% confidence interval [CI]: 17.2–19.5); and at least two types by 5.1% (95% CI: 4.4–5.8). Women relying on the public health sector, and those whose childbirths were via cesarean section with previous labor, had the highest risk, with approximately a three- and two-fold increase in risk, respectively.

**Conclusions:**

Our study showed that the occurrence of disrespect and abuse during childbirth was high and mostly associated with payment by the public sector and labor before delivery. The efforts made by civil society, governments and international organizations are not sufficient to restrain institutional violence against women during childbirth. To eradicate this problem, it is essential to 1) implement policies and actions specific for this type of violence and 2) formulate laws to promote the equality of rights between women and men, with particular emphasis on the economic rights of women and the promotion of gender equality in terms of access to jobs and education.

## Plain English summary

Violence against women in health-care institutions is an emergent and global problem. In our study, we describe the prevalence of disrespect and abuse of women during childbirth among women delivering members of the 2015 Pelotas birth cohort, a large population-based study in which 4275 women participated. We also investigated the factors responsible for such disrespect and abuse. Information relating to disrespect and abuse during childbirth was obtained by household interview 3 months after delivery and included verbal and physical abuse, denial of care and invasive and/or inappropriate procedures. Approximately 10% of these women reported having experienced verbal abuse, 6% denial of care, 6% undesirable or inappropriate procedures and 5% physical abuse. At least one type of disrespect or abuse was reported by 18.3% of mothers; and at least two types, by 5.1%. Women relying on the public health sector, and those whose childbirths were via cesarean section with previous labor, presented the highest risk, with approximate three and two-fold increases in risk, respectively. Our study showed that the occurrence of disrespect and abuse during the process of childbirth was high and mostly associated to payment by the public sector and labor before delivery. To eradicate this problem, it is essential to implement policies and actions specific for this type of violence and to promote the equality of rights between women and men, with particular emphasis on women’s economic rights and the promotion of gender equality with respect to access to jobs and education.

## Background

Access to secure and high-quality sexual and reproductive health services is an essential right for women as these services can make an important contribution towards the reduction of maternal morbidity and mortality rates [1]. In Brazil, some major changes have occurred recently in relation to access to reproductive, maternal and child health services. Attending at least one antenatal care appointment is practically universal (98%), and 81% of all pregnant women have at least five visits [2]. Hospital deliveries are also practically universal, accounting for 98% of all births in this country [2]. Nevertheless, in spite of such impressive figures, the quality of care is not consistent across different population groups thus creating a significant matter for concern [2].

Every woman has the right to quality healthcare which is dignified, respectful, violence-free and free of discrimination. Abuse, negligence or disrespect during the process of childbirth constitute serious violations of fundamental human rights that are recognized internationally [3–5]. In Brazil, a number of public policies have been implemented over recent years aiming towards the humanization of pregnancy and delivery care, such as the Program for Humanization of Antenatal and Delivery Care implemented by the Ministry of Health in 2000 [6]. Furthermore, by law, all women are entitled to be accompanied by a person of their choice during antenatal appointments and hospitalization for delivery, including labor, childbirth and postpartum care [7].

Various forms of mistreatment and abuse of women during childbirth have been described in the literature, including physical, psychological or verbal abuse, humiliation, discrimination, denial of care and the implementation of contraindicated or improper procedures. These can occur at the level of the relationship between women and their healthcare professionals, or at the level of the healthcare service or system [1, 8, 9].

Studies published during the 1990s were the first to raise attention to the issue of obstetric violence [10–12]. Several studies conducted in Brazil [13–15], and elsewhere [1, 8, 9], have indicated that large numbers of women have become victims of this type of violence. However, violence against women during childbirth has now gained wide visibility and is acknowledged as an emerging problem, with studies reporting a prevalence of 11% to 98% [8, 9, 13, 14, 16–22]. Some of these studies have evaluated the potential relationship between disrespect or abuse during childbirth and the factors that might be associated with this practice, such as education, income, type of labor and ethnicity. However, none of the results arising from such studies have been consistent [14, 19, 23, 24], thus reinforcing the need for further studies. Most of these previous studies have relied upon qualitative methods. In particular, there are very few population-based studies, which may provide frequency estimates and identify vulnerable groups, in terms of individual and contextual factors associated with violence during childbirth [8, 9, 13].

The objective of the present study, therefore, was to evaluate the incidence of disrespect and abuse of women during the process of childbirth and to investigate factors that might be associated with this practice. Our study was based on a large population of women who gave birth in 2015 in the city of Pelotas, Brazil.

## Methods

The study was based upon the 2015 Pelotas birth cohort [25], a population-based study that recruited all children born to mothers who were resident in the urban area of the municipality of Pelotas. This city is located in the extreme south of Brazil, with an urban population of 316,000 inhabitants and an annual per capita gross domestic product of approximately US$ 5390.

Women delivering in all of the city’s hospitals between January 1st 2015 and December 31st 2015 were recruited and interviewed, and a follow-up home visit was carried out when each baby was 3 months of age plus or minus 7 days. Data were collected through structured questionnaires applied by trained interviewers; detailed methodology can be found in an earlier publication [25].

There are several terminologies relating to violence against women during the process of childbirth, including obstetric violence and mistreatment [26]. In our study we used the phrase “disrespect for and abuse of women during the process of childbirth”, as used by the World Health Organization (WHO) [27]. At the three-month follow-up interview, four questions were used to identify disrespect and abuse: verbal abuse (“Has any professional been rude to you, cursed you or yelled at you, humiliated you or threatened not to attend to you?”); denial of care (“Has any professional refused to give you anything that you asked for, such as water or painkillers?”); physical abuse (“Has any professional ever pushed, hurt, beat or held you strongly or conducted any examinations rudely or disrespectfully?”) and disrespect regarding invasive and/or inappropriate procedures (“Has any professional ever conducted any procedure against your will, without explaining the need to conduct it?”). For all questions, women were asked to consider their entire hospitalization period, from the time when they arrived until hospital discharge. Each question was coded as “yes” or “no”, and positive responses were added to create a score ranging from zero to four. To identify risk factors, two dichotomous variables were constructed: a report of one or more types of disrespectful treatment or abuse, and a report of two or more types.

Socioeconomic and demographic characteristics were collected during the perinatal interview, including mother’s age at delivery (up to 19 years, 20 to 29 years, 30 to 39 years or 40 years and over), self-reported skin color (white, brown or black), marital status (with or without partner), maternal education (up to 4 years, 5 to 8 years, 9 to 11 years or 12 years or more), family income (measured in Brazilian reais and divided in quintiles), type of payment for childbirth (through the public Brazilian National Health System or other, including private care and private insurance) and type of childbirth (cesarean section after the onset of labor, cesarean section before the onset of labor or vaginal delivery). Women who underwent cesarean section and answered “yes” to the question “Did you have regular contractions (at least one every ten minutes) before the cesarean section?” were considered as having gone into labor.

Quality control measures included repeating a short version of the perinatal and three-month interview to 10% of the women by means of a telephone call, or through a home visit if the woman could not be contacted by phone.

Stata 13 software was used to conduct statistical analyses (StataCorp. 2013. *Stata Statistical Software: Release 13*. College Station, TX: StataCorp LP) and the database that generated the results of this study can be accessed by contacting the corresponding author. Firstly, descriptive analyses of the characteristics of recruited women were performed and the frequencies of each type of violence, disrespect or abuse were obtained. Poisson regression was then used to conduct crude and adjusted analyses of risk factors. Poisson regression represents a more suitable alternative than logistic regression for this type of analysis because the odds ratios are overestimated in comparison to prevalence ratios, especially when estimates are low [28]. Variables that presented *p*-values lower than 0.20 in the crude analysis were then included in adjusted analyses, following a hierarchical causal model. Age, skin color, marital status, educational level and income were included as variables in the first level of this analysis. Variables relating to the type of financing used to cover hospitalization for childbirth was included in the second-level analysis while the type of childbirth was included in the third-level analysis. Because poverty and age can be strongly associated with outcome, we decided to retain family income and women’s age in all models, regardless of the *p*-value in an effort to minimize potential bias.

This study was approved by the Research Ethics Committee of the Higher School of Physical Education of the Federal University of Pelotas (CAAE 26746414.5.0000.5313). All the women provided informed written consent.

## Results

A total of 4333 women gave birth in the city’s hospitals, of whom 4275 (98.7%) agreed to participate in this study. During the three-month follow-up period, 4110 interviews were conducted (a follow-up rate of 97.2%). Of these, 4087 interviews (95.6%) took place with the biological mothers.

Table 1 presents the socioeconomic, demographic and delivery characteristics of our study population. Nearly half of the mothers were aged 20 to 29 years (47%), and most (71%) classified their skin color as white (71%). The majority of women lived with a spouse (86%), had at least 9 years of education (65%), relied on the national health service (68%) and had cesarean sections (65%).

**Table 1:** Associated factors to disrespect or abuse during the childbirth in 2015 Pelotas Birth Cohort (*n* = 4275)

Characteristic	Number	Percent	At least one type of disrespect or abuse		At least two types of disrespect or abuse	
			Crude PR (95%CI)	Adjusted PR (95%CI)	Crude PR (95%CI)	Adjusted PR (95%CI)
Age (full years)			*< 0.001*	*< 0.001*	*< 0.056*	*0.646*
< 20	622	14.6	1.96 (1.20–3.20)	1.77 (1.06–2.96)	1.94 (0.71–5.34)	1.55 (0.55–4.41)
20 to 29	2017	47.2	1.50 (0.93–2.43)	1.39 (0.85–2.29)	1.57 (0.59–4.19)	1.36 (0.51–3.64)
30 to 39	1510	35.3	1.12 (0.68–1.82)	1.12 (0.68–1.84)	1.17 (0.43–3.17)	1.12 (0.41–3.03)
40 to 49	125	2.9	1	1	1	1
Skin color (self-reported)			*0.139*		*0.984*	
White	3024	70.8	1	–	1	–
Brown	551	13	1.11 (0.92–1.34)		1.03 (0.69–1.54)	
Black	667	15.7	1.11 (0.97–1.28)		1.03 (0.71–1.48)	
Marital status			*0.056*	*0.603*	*0.570*	–
With partner	3667	85.8	1	1	1	
Without partner	607	14.2	1.18 (1.00–1.41)	1.05 (0.87–1.26)	1.11 (0.77–1.60)	
Maternal education (years completed)			*< 0.001* ^*+*^	*0.595* ^*+*^	*0. < 001* ^*+*^	*0.101* ^*+*^
< 5	391	9.2	1.38 (1.09–1.76)	1.06 (0.80–1.40)	2.16 (1.34–3.47)	1.77 (0.99–3.13)
5 a 8	1095	25.6	1.43 (1.20–1.71)	1.02 (0.82–1.28)	1.73 (1.17–2.54)	1.41 (0.88–2.26)
9 a 11	1457	34.1	1.24 (1.04–1.47)	1.00 (0.83–1.21)	1.67 (1.16–2.41)	1.50 (1.01–2.25)
> =12	1330	31.1	1	1	1	1
Family income			*< 0.001*	*< 0.001*	*< 0.001*	*0.403*
1° quintile (poorest)	796	19.8	1.65 (1.29–2.11)	1.37 (1.02–1.82)	1.66 (1.02–2.70)	1.11 (0.63–1.96)
2° quintile	807	20.1	1.97 (1.56–2.50)	1.71 (1.31–2.23)	1.71 (1.06–2.77)	1.21 (0.72–2.04)
3° quintile	804	20.0	1.31 (1.01–1.69)	1.18 (0.89–1.55)	1.13 (0.67–1.91)	0.82 (0.47–1.41)
4° quintile	895	22.3	1.55 (1.21–1.98)	1.44 (1.12–1.85)	1.50 (0.93–2.43)	1.23 (0.75–2.02)
5° quintile (richest)	714	17.8	1	1	1	1
Type of payment for delivery care			*< 0.001*	*< 0.001*	*< 0.001*	*< 0.001*
Private care or private insurance	1302	31.7	1	1	1	1
Public sector	2801	68.3	1.88 (1.59–2.22)	1.71 (1.40–2.09)	2.98 (2.01–4.41)	3.23 (2.07–5.03)
Type of labor			*< 0.001*	*< 0.001*	*< 0.001*	*< 0.001*
C-section^a^ after labor onset	1594	37.3	1	1	1	1
C-section^a^ before labor onset	1189	27.8	1.67 (1.41–1.98)	1.45 (1.21–1.73)	2.79 (1.89–4.12)	2.24 (1.50–3.34)
Vaginal delivery	1488	34.9	1.63 (1.38–1.92)	1.22 (1.02–1.46)	2.83 (1.95–4.13)	1.84 (1.22–2.76)

^a^Cesarean section. The numbers in italic are the *p*-values

Table 2 describes the prevalence of each type of violence, and the disrespect and abuse scores. Approximately 10% of the mothers reported having experienced verbal abuse, 6% denial of care, and 5% reported physical abuse. In addition, 6% reported undesirable or inappropriate procedures without an explanation of why it was being conducted. The occurrence of at least one type of disrespect or abuse was reported by 18.3% of mothers (95% confidence interval [CI]: 17.2–19.5), and of at least two types by 5.1% (95% CI: 4.4–5.8).

**Table 2:** Prevalence of disrespect and abuse against women during childbirth, in 2015 Pelotas Birth Cohort

Disrespect or abuse	N	%	95%CI
Verbal abuse	378	9.3	8.4–10.2
Denial of care	240	5.9	5.2–6.6
Physical abuse	183	4.5	3.9–5.2
Invasive and/or inappropriate procedures	236	5.8	5.1–6.5
Score of disrespect and abuse			
None	3338	81.6	80.5–82.8
1 type	542	13.3	12.3–14.3
2 types	141	3.4	2.9–4.1
3 types	51	1.3	1.0–1.6
4 types	15	0.4	0.2–0.6

Regarding the associations between the occurrence of at least one type of disrespect or abuse and socioeconomic, demographic, and delivery factors (Table 1), a higher prevalence ratio was detected among young mothers in the second income quintile, in the public sectors, and when a cesarean section was carried out after the onset of labor. When factors associated with the occurrence of at least two types of disrespect or abuse were evaluated, mothers relying on the public health sector, and those whose childbirths were via cesarean section with previous labor, presented the highest risk, with an increased risk of three and two-fold, respectively (Table 1).

## Discussion

Approximately 18% of women who delivered babies in the city of Pelotas during 2015 were victims of at least one type of disrespectful or abusive treatment during the process of childbirth. This result is similar to the findings of a previous study conducted in Kenya [23], in which 20% of women reported experiencing some type of mistreatment or abuse by healthcare professionals during labor. In a study of eight institutions in a rural area in Tanzania, Kruk et al. [21] also reported a prevalence of approximately 20%, which was slightly lower than another study conducted in the same country [18]. In the latter study, there was no significant difference in abuse when compared between human immunodeficiency virus (HIV)-positive and -negative women. These findings are in contrast with those from a study conducted in Nigeria, where Okafor et al. [19] reported that 98% of women experienced some form of disrespectful or abusive treatment among the 460 puerperal women interviewed at a child immunization clinic. The almost universal prevalence reported by that study could be explained by a detailed evaluation of the different types of disrespect and abuse, for example, episiotomy, augmentation of labor, shaving of pubic hair, sterilization, cesarean delivery and blood transfusion and aspects relating to the payment of hospital fees. The specific form of disrespectful or abusive treatment that contributed most to the observed high levels of prevalence was non-consent care, at a prevalence of 55% [19].

Some studies have reported disrespectful or abusive treatment during childbirth in Latin America. In Venezuela, a hospital study using a self-applied confidential questionnaire showed that 49.4% of women giving birth reported having experienced some form of inhumane treatment from healthcare professionals [24]. In a survey across three Mexican hospitals, 11% of mothers reported some form of mistreatment by healthcare professionals during the process of childbirth [29]. In Brazil, a study conducted in both public and private hospitals across 25 states showed that 25% of women reported having experienced some type of obstetric violence [15]. The wide range of prevalence evident in these studies, from 11% to 98%, may be related to different definitions of obstetric violence, but nevertheless there is no question that the problem is a common one.

Our present results showed that younger age, family income, type of hospitalization system for childbirth and type of childbirth were associated with the incidence of abuse or disrespect during the process of childbirth.

Several studies have been published regarding the relationship between disrespect or abuse during childbirth and a range of associated factors; however, these previous studies yielded inconsistent results. Abuya et al. [23] found no association of any type between disrespect/abuse and maternal age, educational level, marital status and the presence of family/friends, but did show an association between parity and socioeconomic status and between detention for lack of payment and bribery requests, factors that were not included in our present analyses. In another study, Terán and colleagues [24] failed to find an association between dehumanizing treatment and educational level, but did identify a positive association with extreme age groups (adolescents and older women). These authors also showed an association between non-consented procedures with educational level and the type of childbirth. Kruk et al. further demonstrated that disrespect or abuse were associated with higher educational level, higher parity, poverty, cesarean section and depression; however, these authors failed to find an association with age, marital status and health facility factors. In another study, Okafor and colleagues [19] found no association between disrespectful or abusive care during childbirth and any other factor analyzed (maternal age, tribe, marital status, educational status and parity).

A study referred to as ‘Birth in Brazil’ (Nascer no Brasil), a nationally representative population-based study in which a total of 15,688 women were interviewed by telephone during the postpartum period, showed a higher chance of verbal, psychological or physical violence among women who went into labor (odds ratio [OR]: 1.79; 95% CI: 1.28–2.52), and a lower chance among those whose childbirth was privately financed (OR: 0.41; 95% CI: 0.30–0.56) and those who were accompanied by a family member throughout the childbirth process. There was no association between violence and skin color, socioeconomic position, educational level, maternal age or the type of delivery (categorized into vaginal and cesarean section only) [14].

In the Brazilian private or supplementary healthcare network, 90% of childbirths occur by means of cesarean section; of these, 78% occur without the pregnant women having gone into labor [22]. Considering that there is a greater likelihood of abuse and disrespect among women who go into labor, it might be expected that there would be a lower occurrence among those hospitalized in the private system.

Despite international recommendations suggesting that the proportion of cesarean sections should not exceed 15% [30], the levels of surgical deliveries in Brazil have reached epidemic levels and account for 55% of childbirths [22]. This reflects an over-medicalization of birth which has unfortunately accompanied public health achievements such as increased access to prenatal care and a higher proportion of childbirths in healthcare institutions [2].

The greater occurrence of violence among women who went into labor, along with the medicalization of childbirth, may be indicative of a lack of training among medical professionals in carrying out vaginal deliveries. In general terms, Brazil has adopted a highly medicalized model for obstetric care, which uses high levels of technology and little participation from the women who receive this care. During the process of childbirth, the doctor is an authority figure who holds knowledge and power, and is the protagonist of the process. In this, the woman in labor acts merely as an assistant who must obey all medical instructions and accept the procedures that are often imposed on her. In this context, disrespect and/or abuse would consist of the abusive use of power when the authority of a doctor, also represented by the team of other professionals that act under his/her orders, is directly or indirectly challenged by “disobedience”, resistance or questioning [13, 31, 32]. It is also important to consider the fact that disrespect and abuse of women during the process of childbirth is permeated by issues of medicalization relating to their bodies and gender, and reflects the depreciation of their sex and the normalization of violence against women [20, 33].

Women within the Brazilian civil society have become mobilized to fight for dignified and respectful care during pregnancy and childbirth. The movement referred to as “Delivery through Principles – Women’s Network for Active Maternity” (Parto do Princípio – Mulheres em Rede pela Maternidade Ativa), acts to promote women’s autonomy and defend their sexual and reproductive rights, especially regarding awareness during maternity, and has denounced institutional violence during pregnancy and childbirth care to policy makers [34]. However, despite these initiatives, and the implementation of public policies such as the Program for Humanization of Labor and Childbirth, disrespectful treatment and abuse of women during childbirth still occur frequently.

In 2014, the magnitude and severity of this issue at a global level led the WHO to publish a statement [1] proposing actions to eliminate this form of violence. This statement proposed to provide greater support for research and actions, to develop programs to improve the quality of maternal healthcare, to emphasize the right for women to receive dignified and respectful care during pregnancy and childbirth and to monitor data relating to this issue and to involve all stakeholders in a concerted effort to improve the quality of care and eliminate disrespectful practices.

One possible limitation of the present study may lie in the under-reporting of disrespectful or abusive treatment, which may have reduced the magnitude of our estimates and diluted the associations observed. To minimize information bias and to avoid embarrassment and the fear of possible retaliation by the hospital or healthcare professionals, information relating to disrespect and abuse during childbirth were obtained during the three-month follow-up period. It is also important to consider the influence of women’s perceptions on their reports. For example, women who had received lower levels of education and were in lower socioeconomic positions may have tended not to notice abuse because they may have considered this to be part of the normal process. On the other hand, women who presented with higher educational levels and, probably, higher levels of information relating to their rights, may have noticed more subtle forms of violence. Another important limitation is the fact that there was no individualized information on which professionals perpetrated the abuse, or whether they were doctors or nurses. Therefore, in our discussion, we considered doctors to be hierarchically superior and responsible for the team and for the decisions made during the process of childbirth.

Considering that pregnant women are in a situation of great vulnerability, and that the agents of violence against them are the ones that should be providing comprehensive care, the proportion of women who experience disrespect and abuse during the process of childbirth is absurd and unacceptable. It seems that the subjective “judgment” of permitting professionals to adopt unacceptable behavior is more tolerated in healthcare services, particularly public services. It is therefore fundamental to recognize, within the healthcare model, that obstetric care has become too medicalized and that the ethical background of the professionals involved needs to be reviewed.

## Conclusion

The occurrence of disrespect and abuse during the process of childbirth in our study was high and mostly associated to payment by the public sector and labor before delivery. The efforts made by civil society, governments and international organizations are not yet sufficient to restrain institutional violence against women in childbearing. To eradicate the problem, it is essential to 1) implement policies and actions specific for this type of violence and 2) formulate laws to promote the equality of rights between women and men, with particular emphasis on women’s economic rights and the promotion of gender equity with respect to access to jobs and education. In this way, it is possible to alter the social norms that strengthen and perpetuate social models that can ultimately lead to a lack of autonomy in women, and exert control over processes which relate to their own bodies and lives, and to the various forms of violence against women [29].
